# 

**Published:** 1905-08-19

**Authors:** 


NOTICE TO CORRESPONDENTS.

All MSS., Letters, Books for Review, and other matters intended for the Editor should be addressed The Editor, "The Hospital," The Hospital Buildings, 28 & 29 Southampton Street, Strand, W.C.

The Editor cannot undertake to return rejected MS., even when accompanied by stamped directed envelope.

**Notice to Advertisers and Subscribers.**

All Advertisements, Orders for Copies of The Hospital, and Business Communications should be addressed to **THE MANAGER (Not to the Editor),** The Hospital, 28 & 29 Southampton Street, Strand, London, W.C.

The Cost of Subscription, post free, is at follows:—Per week, 3½d.; per quarter, 3s. 3d.; per half-year, 6s. 6d.; per annum, 13s. Foreign and Colonial:—Per annum, 19s. 6d., payable, in all cases, in advance.

**NOTICE.—Vol. XXXVII. of "The Hospital" October 1904 to March 1905), in handsome cloth binding, will shortly be on sale at the office of this Journal, price 10s. 6d. Cases for binding the Numbers contained in this Volume 2s. Index to Vol. XXXVII., 3½d., post free.**

**The Monthly Part for August is now ready. Price 1s., by post, 1s. 3d.**

### BOOKS RECEIVED.

H. K. Lewis.

"The Child's Diet." By J. Sadler Curgenven.

J. Wright and Co.

"Golden Rules of Psychiatry." By Dr. James Shaw.

### MEDICAL APPOINTMENTS.

**William James Anderson,** L.R.C.P. & S.Edin., L.F.P.S. Glasg., District Medical Officer and Public Vaccinator by the Helston (Cornwall) Board of Guardians, and Certifying Surgeon under the Factory and Workshop Act for the Helston District of the County of Cornwall. **J. Blumfeld,** M.D.Cantab., Honorary Anæsthetist to St. Mary's Hospital, Paddington, W. **Victor Bonney,** M.S., M.D., B.Sc.Lond., F.R.C.S., M.R.C.P., Emden Research Scholar to the Cancer Investigation Department of the Middlesex Hospital. **F. G. Bushnell,** M.D.Lond., a Vice-president of the Devon and Cornwall Sanatorium for Consumptive Poor. **Edward Arthur Eckersley,** M.B., C.M., Clinical Assistant to the Hospital for Diseases of the Skin, Blackfriars, S.E. **Thomas Evans,** M.D., Clinical Assistant to the Hospital for Diseases of the Skin, Blackfriars, S.E. **E. Fielden,** M.B., M.S.Durh., Certifying Surgeon under the Factory and Workshop Act for the Bracknell District of the County of Berks. **E. M. Goldie,** M.D.Edin., Visiting Surgeon to the Royal Merchant Seamen's Orphanage, Snaresbrook. **J. M. Pooley,** M.R.C.S., L.R.C.P.Lond., Medical Officer and Public Vaccinator for the Nettlebed District of the Henley Union. **Frank Robinson,** M.D., D.P.H., Deputy Medical Officer of Health of the City of Westminster. **H. B. Roderick,** M.D., B.C.Cantab., Clinical Assistant to the Chelsea Hospital for Women. **Arnold Winkelried Williams,** M.B., C.M., D.P.H., R.C.P.S., Clinical Assistant to the Hospital for Diseases of the Skin, Blackfriars, S.E.

*Published by*
THE SCIENTIFIC PRESS, LTD.
28 & 29 Southampton Street,
Strand, London, W.C.

### The Book for the C.M.B. Exam.

### A COMPLETE HANDBOOK OF MIDWIFERY.

6/- net, 6/4 post free.

By J. K. WATSON, M.D.,

Author of "A Handbook for Nurses."

143 Illustrations.

6/- net, 6/4 post free.

This book has been compiled in accordance with the requirements of the Central Midwives Board, and will prove not only the best text-book for those entering for the next exam., but is so complete as to be of greatest value to the Qualified Midwife for immediate reference in cases of emergency.

### A Valuable Work for Practising Midwives.

### A PRACTICAL HANDBOOK OF MIDWIFERY.

6/- net, 6/3 post free.

By FRANCIS W. HAULTAIN, M.D.

New Edition. Illustrated. Bound in Leather.

6/- net, 6/3 post free.

### Handy Guides for the Apron Pocket.

### THE MIDWIVES' POCKET-BOOK.

1/- post free.

By HONNOR MORTEN.
Cloth.

1/- post free.

### NURSING NOTES ON MIDWIFERY AND GYNÆCOLOGY.

2/- net. 2/1 post free.

By SISTER ROSS, Rotunda Hospital.
Cloth.

2/- net, 2/1 post free.

### A Complete Explanation of the Midwives Act.

### HOW TO BECOME A CERTIFIED MIDWIFE.

1/- net, 1/1 post free.

By E. L. C. APPEL, M.B.

Limp Cloth.

1/- net. 1/1 post free.

### A New Work for Midwives and District Nurses.

### SIMPLE SANITATION: The Practical Application of the Laws of Health to Small Dwellings.

Limp cloth, 1/- net, 1/2 post free.

By M. LOANE,
Superintendent of District Nurses, Portsmouth.

Limp cloth, 1/- net, 1/2 post free.

With an Introductory Chapter on Sanitary Legislation by Dr. A. Mearns Fraser, Medical Officer of Health, Portsmouth.

This work should prove of great practical assistance to Private and District Nurses, as it contains Chapters on Sanitary Legislation, Water, Air and Ventilation, Drainage and Disposal of Refuse, Household Management, Personal Hygiene, Disinfectants, &c.

***Complete catalogue post free on application to***

**The Scientific Press, Ltd.**

Publishers of Nursing Text-Books, Charts, and Case-Books, of all descriptions.

28 & 29 SOUTHAMPTON STREET,
STRAND, LONDON, W.C.

